# Validation and psychometric properties of the Indonesian version of the Fear of Missing Out Scale in adolescents

**DOI:** 10.1186/s41155-021-00181-0

**Published:** 2021-06-12

**Authors:** Dian Veronika Sakti Kaloeti, Ayu Kurnia S, Valentino Marcel Tahamata

**Affiliations:** 1 Faculty of Psychology, Universitas Diponegoro, Semarang, Indonesia; 2 Graduate Institute of Mind, Brain and Consciousness, Taipei Medical University, Taipei, Taiwan

**Keywords:** Fear of Missing Out Scale, Validation, Psychometric properties, Indonesian, Adolescent

## Abstract

**Background:**

This study’s main purpose was to examine the psychometric properties of FoMOs’ adaptation among the Indonesian adolescents’ population. The second aim was to investigate the concurrent validity of the Indonesian version to provide evidence for the validity. Also, FoMOs’ difference level between demographic variance analyses was performed.

**Method:**

The study involved a cross-sectional online survey design with 638 Indonesian adolescents aged 16–24 (M = 19.08, SD = 14.70). FoMO was measured by a 16-item that has been modified from the original 10-item. Exploratory and confirmatory factor analyses were carried out to study its scores’ evidence of structural validity. Besides, to study its scores’ evidence of convergent, discriminant, and predictive validity concerning other variables such as stress, anxiety, and depression (Depression and Anxiety Stress Scale-21), and general health condition (General Health Questionnaire Scale-12), correlation analyses were conducted. To study the sensitivity, we assessed the effect of sociodemographic and social media use on the scale’s ability to identify the population’s risk to the FoMO by conducting analyses of variance. The Cronbach alpha values (α = .93) indicated that internal consistency of the scale was at an adequate level.

**Results:**

Exploratory factorial analyses revealed adequate adjustment for the new version of the scale showing the three factorial structures. Confirmatory factor analyses showed that the 12-item of Indonesian FoMO had a good fit (χ^2^/df = 289.324/51; goodness-of-fit index (GFI) = 0.928; RMSEA = 0.086; comparative fit index (CFI) = 0.915; normed fit index (NFI) = 0.899; parsimony normed fit index (PNFI) = .695; Tucker–Lewis index (TLI) = 0.890).

**Conclusion:**

This study has shown that the modified 12-item Fear of Missing Out Scale is a valid and reliable instrument for Indonesian adolescents. It showed that the Indonesian version of Fear of Missing Out Scale has adequate psychometric properties to measure Indonesian adolescents’ online behavior.

## Introduction

Indonesia is one of the countries with the fastest growth of social media users globally (Digital Information World, 2018). The same report also reveals that Indonesia has experienced increased social media users by 51% throughout 2018. UNICEF (2014) noted that 80% of children and adolescents in Indonesia are active internet users including social media. People actively choose social media because they would like to satisfy their particular needs such as social interaction, entertainment, information seeking, and sharing (Dolan, Conduit, Fahy, & Goodman, 2016Talaue, AlSaad, AlRushaidan, AlHugail, & AlFahhad, 2018). Apart from their benefits, social media may pose some problems. Some other researchers have also indicated that participation in social media such as WhatsApp, Twitter, and Instagram negatively affects wellbeing, overall mood, and life satisfaction (Burke, Marlow, & Lento, 2010; Przybylski, Murayama, DeHaan, & Gladwell, 2013; Chou & Edge, 2012; Sampasa-Kanyinga & Lewis, 2015). Social media may change the daily habit and induce malicious behavior of the users, like excessive use that results in social media addiction (Liftiah, Dahriyanto, & Tresnawati, 2016). The latter addiction can cause long-term interpersonal relationship and mental health problems, such as severe depression and anxiety symptoms, negative mood, boredom, and loneliness (Chou & Edge, 2012; Edwards, 2017; Rao, 2017). This condition can also trigger the individual’s fear of missing out (FoMO) (Milyavskaya, Saffran, Hope, & Koestner,  2018).

This research uses the definition of FoMO introduced by Przybylski et al. (2013). The terminology of fear of missing out (FoMO) was coined by Przybylski et al. (2013). FoMO is defined as “a pervasive apprehension that others might be having rewarding experiences from which one is absent” and “a desire to stay continually connected with what others are doing” (Przybylski et al., 2013). This typically refers to preoccupation with constantly connecting to social media due to the need not to miss something or other rewarding activities. This condition can describe the emergence of social anxiety feelings towards uncertainty on satisfying topic alert that might be missed while not in online activities.

FoMO is associated with self-determination theory characterized by the desire to continually stay connected with what others are doing (Przybylski et al., 2013). Interestingly, Przybylski et al. (2013) avoided using the terminology of “addiction” for describing the correlation of social media usage but instead preferred to use the term “Fear of Missing Out (FoMO).” It is not about missing the mobile phone, but it is about the fear of not being part of what friends are doing.

Currently, FoMO as a form of addiction is still being debated. In several studies, it is stated that FoMO is a type of addiction in new technologies (Przybylski et al., 2013Tomczyk & Selmanagic-Lizde, 2018). Smartphone addiction arises from the fear of being left behind on something that happens on social media (Przybylski et al., 2013). However, to date, FoMO has not been classified as a disease in the DSM or ICD (Griffiths, Kuss, & Demetrovics, 2014). Another study explains that FoMO is only related to problems that arise from smartphone use and one factor in addiction formation. Griffiths et al. (2014) emphasized that FoMO is a risk factor for social media addiction. Hosgor et al. (2017) stated that FoMO is related to the number of social media accounts and the frequency in controlling these accounts. In the research of Alutaybi, Al-Thani, McAlaney, and Ali (2020), FoMO fulfills several symptoms in the adaptation process, especially from social media and withdrawal signs.

FoMO is a reasonably new construct involving the person’s reluctance to miss important information and social events (Przybylski et al., 2013). FoMO is a construct concerning unmet social needs and is conceptualized from depression and social anxiety (Oberst, Wegmann, Stodt, Brand, & Chamarro, 2017; Wegmann, Oberst, Stodt, & Brand, 2017). FoMO also involves negative expectancies and cognitions, which play a role in problematic internet use (Wegmann et al., 2017). Individuals with higher levels of FoMO will be more alert to smartphone notifications and will have weakened concentration (Duke & Montag, 2017). Furthermore, FoMO causes continuous psychological changes in which individuals’ tendency to make use of SNS increases (Przybylski et al., 2013). In the research of Trepte and Reinecke (2013), individuals who fear of being excluded tend to have less control in their online life by promoting themselves by editing and updating the content on their profiles. Furthermore, Kramer, Hoffman, and Eimler (2015) confirmed a relationship between the need to be connected or connected and social media behavior. Other recent studies found that FoMO mediated the relations between psychopathology and inappropriate smartphone use (Wegmann et al., 2017; Elhai, Levine, Dvorak, & Hall, 2018; Oberst et al., 2017). Moreover, adolescents’ mental health problems will have a substantial impact on their subsequent developments (Ediati, 2015) and increase emotional problems (Kaloeti, 2016).

FoMO research has been developed in several countries with large sample size. The fear of missing out among students at Kuwait University is high and affects the distraction of attention and absence from learning activities. This study also reveals no significant relationship between FoMo and student academic background (Al-Furaih & Al-Awidi, 2020). Another study conducted in Bosnia and Herzegovina by Tomczyk and Selmanagic-Lizde (2018) showed that 30% of young internet users in Bosnia and Herzegovina are at risk of developing FoMO. In addition, research on adolescents in Israel shows that high levels of conflict in the family are related to problems with internet use and time spent on social media (Sela, Zach, Amichay-Hamburger, Mishali, & Omer, 2020). This condition results in higher levels of depression and a stronger FoMO (Sela et al., 2020).

Przybylski et al. (2013); Abel, Buff, and Burr (2016); Metin, Pehlivan, and Tarhan (2017); and Riordan et al. (2018) have recently developed a single measurement to assess the FoMO. The most common and popular measure is the four-factor and single-factor FoMO scale (henceforth the FoMOs) (Przybylski et al., 2013), which is relatively simple and convenient. It contains only ten items. Numerous studies have found favorable psychometric properties of FoMO and adapted previously to Turkish (Can & Satici, 2019), Arabic (Al-Menayes, 2016), Spanish (Gil, Oberst, Del Valle, & Chamarro, 2015), and English (Perrone, 2013). The Arabic version of the FoMOs showed a two-factor structure with .82 and .72 Cronbach alpha values. In contrast, the Turkish, Spanish, and English versions supported the original one-factor design with .81, .85, and .93 Cronbach alpha values, respectively. In Indonesia, the adaptation and psychometric properties of FoMO were done in adolescents (Syahniar et al., 2018), but involved only 30 participants using the original 10-item FoMOs so that further research is required following the characteristics of Indonesian adolescents for the justification of the validity of the scale. Therefore, this study’s primary purpose was to examine the psychometric properties of FoMOs’ adaptation among the Indonesian adolescents’ population. The second aim was to investigate the concurrent validity of the Indonesian version to provide evidence for the validity. In addition, the FoMOs’ difference level between demographic variance analysis was performed.

## Research method

### Participants

A cross-sectional online survey was conducted on 638 adolescents in Indonesia. The procedure for selecting participants was carried out by purposive sampling by distributing the scale to participants who match the inclusion criteria through the google form application. The inclusion criteria for research participants were Indonesian social media users at the ages ranging from 16 to 24, due to almost all adolescents in the age range use social networks and the vast majority owns a smartphone (Pitchforth et al., 2018; Royal College of Psychiatrists, 2010).

### Data collection process

A cross-sectional online survey was carried out among participants. This study was approved by the Research Ethics Committee of the first author’s institution and complied with the ethical standards for research involving human subjects. An online survey questionnaire was distributed to the participants. Before the survey was administered, participants were provided with informed consent that their answers would remain anonymous. Participation is voluntary and not receiving credits or incentives.

The questionnaire contained sociodemographic information, 16-item Fear of Missing Out Scale (FoMOs) that has been modified and translated into Bahasa Indonesia, 12-item General Health Questionnaire (GHQ-12), and 21-item Depression Anxiety Stress Scale (DASS-21). The sociodemographic information consisted of personal subject information such as age, gender, ethnicity, the most used social media, and duration of accessing social media in 1 day.

### Measurements

#### Demographic questionnaires

The sociodemographic questionnaires consisted of personal subject information such as sex, age, gender, ethnicity, amount of monthly parent’s income, the most used social media, and duration of accessing social media in 1 day.

##### Fear of Missing Out (FoMO)

The FoMO scale is the self-reported degree of fear of missing out, consisting of sixteen-item and four-factor (missed experience, compulsion, comparison with friends, and being left out) conducted by Przybylski et al. (2013). We use the original 10-item FoMO scale (Przybylski et al., 2013), plus six additional items created to ensemble the online behavior of Indonesian adolescents (e.g., “When I buy viral things, I will upload photos of those things”; “I always check my friends’ social media when they are on holiday”). The overall scale including 16 items, scored on a five-point Likert scale (1 = disagree, 5 = agree), was used. The Cronbach α coefficient of the scale for this study was .90.

Before data collection, the FoMO instruments used in this study were adapted and translated using guidelines from Beaton, Bombardier, Guillemin, and Ferraz (2000). In the measurement translation, two psychologists performed forward translation. Next, the Bahasa Indonesia version was back-translated by a native language expert who had not seen the original English version. For this stage, the consensus in terms of grammar, conceptual equivalence, and semantic was reached. The pre-final version of Bahasa Indonesia measures was reviewed and approved, which included a panel of a psychologist and language expert. This version was tested on ten adolescents. They were asked if any terms and sentences were unclear or difficult to understand. The results have shown that the instruments were very easy to complete and very clearly understood by the adolescent participants. Thus, the reliability and validity of the final instruments were tested.

##### General Health Questionnaire

The General Health Questionnaire (GHQ-12) assessing the severity of the mental problem consists of twelve items that were translated and adapted in Bahasa conducted by Nurwanti and Hidayat (2013). Each item was rated on a bimodal scale (0-0-1-1) start from left to correct answer choice. The score was used to generate a total score ranging from 0 to 12, with higher scores indicating worse conditions (Nurwanti & Hidayat, 2013). The Cronbach α coefficient of the scale for this study was .87.

##### Depression Anxiety Stress Scale

The Depression Anxiety Stress Scale (DASS-21) is a set of three self-report scales designed to measure the emotional states of depression, anxiety, and stress. DASS-21 was translated and adapted in Bahasa conducted by Oei, Sawang, Goh, and Mukhtar (2013). Each of the three DASS-21 scales contains seven items, and each item was rated on a Likert scale (0= did not apply to 3= applied very much). To calculate the final score, scores on the DASS-21 need to be multiplied by 2 (Oei et al., 2013). The Cronbach α coefficient of the scale for this study was .91.

### Statistical analysis

The study conducted structural validity (exploratory and confirmatory factor analysis) based on the 16 items of the FoMO and calculated reliability (internal consistency) by looking at Cronbach’s alpha score. For measuring the external criteria (discriminant validity and convergent validity) of FoMO, this study correlates between the FoMO scale, General Health Questionnaire Scale-12 (GHQ-12), and Depression and Anxiety Stress Scale-21 (DASS-21).

## Results

### Demographic analysis

The demographic analysis encompassed several factors, i.e., sex, age, ethnicity, amount of monthly parent’s income, the most used social media, and duration of accessing social media in 1 day that might impact the young’s fear of missing out hurdles as stated in Table 1. The participants were 209 male (32.7%) and 429 female (67.2%) social media users at the ages ranging from 10 to 24 (M = 19.08, SD = 14.70). The most frequently used social media are WhatsApp (34.79%), LINE (32.28%), and Instagram (20.84%), with the most duration of accessing social media is 4–6 h a day (58.15%).

**Table 1:** Demographic analysis

**Category**	**Total**	
	**Frequency**	**%**
**Sex**		
Men	209	32.7
Women	429	67.2
**Age**		
10–15	4	0.63
16–20	515	80.7
21–24	119	18.65
**Ethnicity**		
Javanese	504	78.99
Non-Javanese	80	12.50
**Amount of monthly parent’s income**		
<1 million IDR	85	13.32
1–3 million IDR	332	52.03
>3 million IDR	221	34.63
**The most used social media**		
YouTube	40	6.26
Instagram	133	20.84
WhatsApp	222	34.79
LINE	206	32.28
Others (Facebook, Twitter)	37	5.79
**Duration of accessing social media in 1 day**		
1–3 h	193	30.25
4–6 h	371	58.15
>6 h	74	11.59

### Demographic associated with FOMO

The T-test analysis between demographic data and FoMO shows that ethnic origin (0.07), the most used social media (0.00), and duration of accessing social media (0.07) show a significant difference in the FoMO level experienced by the participants. More detailed data can be seen in Table 2.

**Table 2:** Demographic data associated with FoMO

Variables	Mean	SD	*df*	*t/F*	*p*-value
Sex					
Men	38.04	10.49	208	0.99	0.32
Women	37.16	10.44	428		
Age					
16–20	36.68	12.30	518	0.58	0.57
20–24	38.22	11.17	118		
Ethnicity					
Javanese	37.35	10.23	503	−1.79	0.07*
Non-Javanese	39.29	11.42	133		
Amount of monthly parent’s income					
<1 million IDR	37.00	8.23	84	2.01	0.13
1–3 million IDR	36.96	10.50	331		
>3 million IDR	38.65	10.88	220		
The most used social media					
YouTube	31.55	8.95	39	6.84	0.00**
Instagram	39.96	10.10	132		
WhatsApp	37.69	10.16	221		
LINE	37.86	11.35	205		
Others (Facebook, Twitter)	36.61	10.20	36		
Duration of accessing social media					
1–3 h	36.31	10.49	192	4.98	0.07*
4–6 h	37.86	10.22	370		
>6 h	40.95	11.20	73		

*Comparison is significant at the 0.1 level (2-tailed)

**Comparison is significant at the 0.01 level (2-tailed)

### Descriptive data on FoMO conditions in participants

Descriptive data show that the study participants mainly experienced low levels of FoMO (35.6%) (Table 3). The number of research participants who experienced FoMO at a high level was 6.9% and at a very high level was 1.9%.

**Table 3:** Descriptive data on FoMO conditions in participants

	Frequency	Percent
Very low	226	35.4
Low	227	35.6
Medium	129	20.2
High	44	6.9
Very high	12	1.9
Total	638	100.0

### Convergent validity and discriminant validity

The Pearson correlation coefficients between the FoMO, DASS-21, and GHQ-12 scale were calculated. Table 4 showed positive correlations between the FoMO, DASS-21, and GHQ-12 which have similar measurement structures (*p* < .01). These results suggest that the FoMO has good convergent validity. However, although FoMO was significantly related to DASS-21 or GHQ-12, a significant correlation could also result from the effect of a larger sample size (*p* < .01).

**Table 4:** Correlation matrix of the study variables

	1	2	3	4	5
1.FoMO	-				
DASS-21					
2. Depression	0.65**	-			
3. Anxiety	0.30**	0.39**	-		
4. Stress	0.35**	0.25**	0.45**	-	
5. GHQ-12	0.18**	0.05**	0.34**	0.14**	-

*Correlation is significant at the 0.05 level (2-tailed)

**Correlation is significant at the 0.01 level (2-tailed)

### Reliability analysis

The internal consistency coefficient of the FoMO was computed. The results showed that Cronbach’s alpha coefficient was .91 for the total scale, and the internal consistency coefficients of the four factors were .69 (missed experience), .69 (compulsion), .77 (comparison with friends), and .75 (being left out). The internal consistency coefficient of the FoMO and the four factors reached acceptable levels in the study samples.

### Exploratory factor analysis of fear of missing out (FoMO)

As a preliminary step, the internal structure of the FoMO was analyzed. A principal component analysis with a varimax rotation was performed on the data to explore the possible structure of the FoMO with Indonesian adolescent participants. The procedure resulted in a stable three-factor solution. An exploratory factor analysis (EFA) with principal axis analysis, promax rotation, and parallel analysis was then conducted to assess the modified 16-item FoMO scale structure. Based on factor loadings, items with low psychometric values were excluded. The EFA concluded with a three factorial twelve-item version; four items (CO13, LO14, LO15, and LO16) were removed from the scale due to low item loading results (< .30).

Nine items from the original FoMO scale were kept: four loadings on factor 1, three loadings on factor 2, and two loadings on factor 3. Two items of original FoMO that were included in being left out are item “It is important that I understand my friends “in-jokes” which was removed and the item “I get anxious when I don’t know what my friends are up to” which was included in factor 1. With these three factors, 62.17% of the variance was explained. The first factor’s items represent missed experiences, explicitly the negative feelings that arise because of not being able to be involved in an activity. The second factor’s items represent compulsion, namely, the behavior of repeatedly checking what was done. The items of the third factor represent comparison with friends, that is, negative feelings that arise from making comparisons with friends and other people. The final FoMOs comprises 12 items with a three-factor structure with a high internal consistency (α = .93) (Tables 5 and 6).

**Table 5:** Exploratory factor analysis: factor loadings for the FoMO, principal components, varimax rotation, eigenvalues, and percentage of explained variance (first version)

No	Items	Factor I	Factor II	Factor III	Factor IV
1	It bothers me when I miss an opportunity to meet up with friends.	.68	--	--	--
2	I get worried when I find out my friends are having fun without me.	.84	--	--	--
3	I feel worried if I don’t know what my friends are doing.	.69	--	--	--
4	I feel upset with my friends when they spend their vacation, and I see their photos on social media while I was not invited.	.85	--	--	--
5	When I miss out on a planned get-together, it bothers me.	.79	--	--	--
6	When I go on vacation, I continue to keep tabs on what friends are doing.	--	.47	--	--
7	When I buy very popular things, I will upload photos of those things.	--	.47	--	--
8	I always check my friends’ social media when they are on holiday.	--	.42	--	--
9	When I have a good time, it is important for me to share the details online (e.g., updating status).	--	.31	--	--
10	Sometimes, I wonder if I spend too much time keeping up with what is going on.	--	.48	--	--
11	I feel anxious to get my friends to have the more valuable meaning of their life.	--	--	.45	--
12	I fear others have more rewarding experiences than me.	--	--	.49	--
13	I get anxious when I see other people getting more “likes” on social media than me.	--	--	.05	--
14	I have a constant desire to know the latest news that is being talked about on social media.	--	--	--	.30
15	When there is a notification from a social media application, I will immediately respond even though I’m doing other activities.	--	--	--	−.28
16	I become anxious when a friend blocks me on social media.	--	--	--	−.87
Eigenvalue		1.08	0.75	3.35	0.42
Explained variance %		4.29	3.60	16.48	2.10
Cumulative %		71.99	81.5	57.21	95.86
Mean ± SD		37.75 ± 10.47

**Table 6:** Exploratory factor analysis: factor loadings for the FoMO, principal components, varimax rotation, eigenvalues, and percentage of explained variance (second version)

No	Items	Factor I	Factor II	Factor III
1	It bothers me when I miss an opportunity to meet up with friends.	.80	--	--
2	I get worried when I find out my friends are having fun without me.	.74	--	--
3	I get anxious when I don’t know what my friends are up to.	.69	--	--
4	I feel upset with my friends when they spend their vacation, and I see their photos on social media while I was not invited.	.68	--	--
5	When I miss out on a planned get-together, it bothers me.	.64	--	--
6	When I go on vacation, I continue to keep tabs on what friends are doing.	--	.76	--
7	When I buy very popular things, I will upload photos of those things.	--	.71	--
8	I always check my friends’ social media when they are on holiday	--	.69	--
9	When I have a good time, it is important for me to share the details online (e.g., updating status)	--	.65	--
10	Sometimes, I wonder if I spend too much time keeping up with what is going on.	--	.59	--
11	I fear others have more rewarding experiences than me.	--	--	.91
12	I don’t feel happy when my friends tell their valuable story of their life.	--	--	.91
Eigenvalue		4.53	1.67	1.27
Explained variance %		37.72	13.87	10.58
Cumulative %		37.72	51.59	62.17
Mean ± SD		37.75 ± 10.48		

### Confirmatory factor analysis of the fear of missing out (FoMO)

**Fig. 1:**
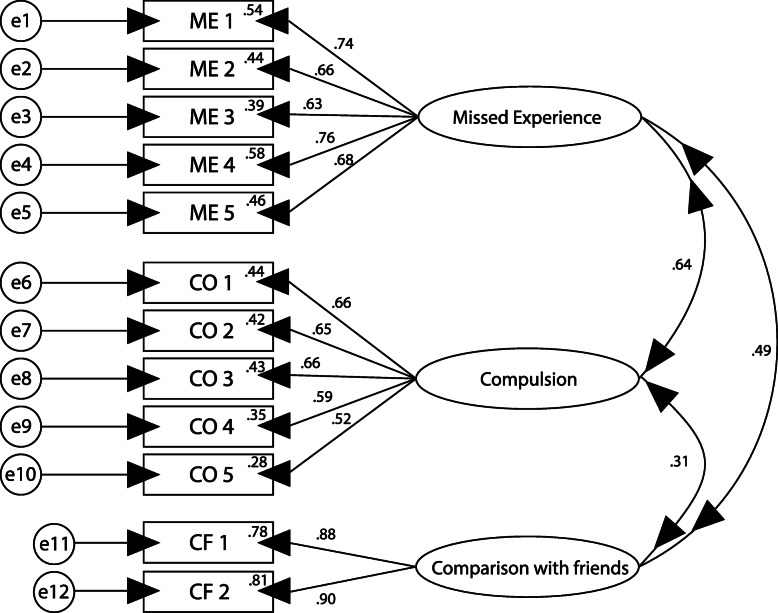
Confirmatory factor analysis of the Indonesian version FoMO

A confirmatory factor analysis was performed via structural equation modeling on the whole data set (Fig. 1). Analyses were performed using maximum likelihood estimation and the robust estimation method.

The three-factor structure had a good fit and was the most parsimonious (Chi-square/df = 289.324/51; GFI = 0.928; RMSEA = 0.086; CFI = 0.915; NFI = 0.899; PNFI = .695; TLI = 0.890). Despite these local dependencies, the factor loadings of the items on the FoMO were high (see Fig. 1: confirmatory factor model). In total, factor loadings exceed .4. Item 3 (missed experienced) (.39), item 9 (comparison with friends) (.34), and item 10 (comparison with friends) (.27) in the sample had somewhat lower loadings (see Table 7).

**Table 7:** Measures of local fit for CFA model

Item	Indicator reliability (IR)	Standard error (SE)	Critical ratio (CR)
ME1	0.54	0.068	13.87**
ME2	0.43	0.079	14.84**
ME3	0.39	0.067	13.78**
ME4	0.57	0.070	16.20**
ME5	0.46	1^1)^	1^1)^
CO1	0.43	0.116	10.68**
CO2	0.42	0.083	10.09**
CO3	0.43	0.106	10.89**
CO4	0.34	0.105	10.57**
CO5	0.27	1^1)^	1^1)^
CF1	0.77	0.072	14.96**
CF2	0.81	1^1)^	1^1)^

Note: **p<0.001, fixed to 1 to ensure identifiability

## Discussion

### Missed experience being the highest contribution than other factors

Young adults tend to act like most people to be considered “normal” by their group (conformity). Conformity refers to changing behavior to “get along” with people, or in other words, to obtain social acceptance. To conform to others is also the nature of young adults in fulfilling their developmental tasks, namely, independence from their parents, gaining intimacy, playing a particular role in society, and committing to a professional career (Benson & Elder, 2011). The presence of the internet and social media is now beneficial for career development by quickly establishing communication. It might help the adolescent to obtain their developmental tasks quickly. The exact mechanism has occurred in this study where the missed experience (factor I) contributes 67.7% and being the highest contribution than other factors. Missed experience refers to the negative emotion due to not being involved or missed a particular activity in social media (Przybylski et al., 2013).

Furthermore, the participants involved in this study are the young adults who are the post-millennial or Z generation and so-called digital natives. They were born and grew up in the digital era; therefore, they have more sensitivity than the previous generation. This condition also yielded the brand new industrial realm, i.e., digital industry. In this digitalized era, people and especially young adults are unconsciously demanded to be engaged in their gadgets and social media. Thus, the digital age provides a different way for young adults to fulfill their developmental tasks (Rue, 2018).

### Eliminated “Being Left Out Factors” and how demographic affecting the result

The results of the study indicate that one factor was eliminated, namely, the being left out factor. Most of the subjects in this study were 18–21 years old (84.4%) and had completed their undergraduate education (89.18%). The more mature and the higher the level of education, the more mature the person’s attitude (Benson & Elder, 2011; Darling-Hammond, Flook, Cook-Harvey, Barron, & Osher, 2019). Young adults tend to solve their problems independently and determine priorities (Benson & Elder, 2011). This encourages individuals to be more neutral when they do not join activities with friends because they consciously understand the consequences of decisions taken.

The result shows that missed experience is the highest contribution than other factors. It can be influenced by the participants’ condition (age, ethnic origin, individual education, and parental education). Javanese is the most ethnic group in this study (78.99%). Another research shows that Indonesia’s Javanese have strong cooperation and family culture (Hermawan & Loo, 2019). This is interpreted as great care so that individuals who do not understand others’ conditions will be judged to have no concern.

### Compulsion in media use is associated with the comparison with friends

In contrast, several recent studies that investigated the connection between problematic internet use, specifically related to excessive use of social media, have found that FoMO provides an endless opportunity for comparison of one’s status and stay continually connected with what others are doing (Alt, 2016; Przybylski et al., 2013). The study of Alt and Boniel-Nissim (2018) also indicated that compulsion in media use is positively associated with friends’ comparison. This condition is also pictured through the results of this study. Compulsion (factor II) and comparison with friends (factor III) contribute 65.6% and 54.7%, respectively, while the comparison with friends (factor III) has remaining two valuable items only. Reer, Tang, and Quandt (2019) argued that FoMO is significantly associated with social comparison orientation. The two variables mediated the psychological wellbeing and social media engagement among adolescents and young adults. The study found that being engaged in social media predicts a higher level of depression, anxiety, and loneliness. This study indicates the high compulsivity of young adults in digital media use, while their status in comparison with other friends is relatively low. Therefore, this condition implies that they are quite a confident generation. They were aligned with this study’s result; Robinson et al. (2018) postulate that social comparison does not predict depressive symptoms when the person has not an inferiority feeling nor negative self-perception. In this case, whether downward or upward social comparison should be considered in predicting young adults’ negative wellbeing; each tends to have different consequences. Some subjects in this study perform their compulsive media use due to their positive outlook toward themselves compared to other social media people.

### FoMO and its relation to type, duration of use of social media, and ethnicity

This research found that WhatsApp, LINE, and Instagram were the most used social media. In contrast to previous studies, adolescents are more likely to use social media on Facebook to communicate with other people (Kuss & Griffiths, 2011). However, Instagram becomes popular because users can post photos and videos in real-time shows (Aprilia, Sriati, & Hendrawati, 2018; Solomon, 2013). Whereas with WhatsApp, they feel more like they have good connections with other people (Lup, Trub, & Rosenthal, 2015; Yus, 2017). Quinn’s (2016) research showed that the desire to maintain social relations which is one factor for adolescents is attached to social media, fulfilling the need for pleasure, closeness, and popularity (Afriluyanto, 2018; Mahendra, 2017; Sakti & Yulianto, 2013).

But on the other hand, this study found that using Instagram tends to experience FoMO. The popularity of Instagram carries the risk of becoming problematic for some of its users (Kuss & Griffiths, 2017). According to Moore and Craciun (2020), individuals with higher fear of missing out are reported to have good attitudes on Instagram, become followers of more Instagram accounts, and have a stronger tendency towards social media addiction. In Munawaroh, Nurmalasari, and Sofyan (2020), it is stated that whenever individuals feel bored or uncomfortable, they will access Instagram. Furthermore, Rahardjo and Mulyani (2020) said that individuals with poor social competence tend to build relationships with the online form. Receiving others’ comments on Instagram can provide positive and meaningful feelings for them (Kircaburun & Griffiths, in Rahardjo and Mulyani (2020)). When individuals feel that Instagram is good for them and can meet their needs, they will be compelled to repeat the pattern of behavior. This study found that accessing social media for more than 6 h per day tends to experience FoMO. A survey conducted by Anwar, Fury, and Fauziah (2020) stated that the higher a person’s tendency towards FoMO, the higher the intensity of social media use. Some of the social media behaviors that can lead to FoMO, such as adolescent feel that they have a high need to use social media, use social media at night, even put their cellphones under their pillow to avoid missed messages at midnight, and skip meals or speed up the duration of meals (Fathadhika & Afriani, 2018). The exciting thing that was found was that the non-Javanese had a higher FoMO level than the Javanese. A study conducted by Hidayati, Syaf, and Hartati (2021) stated that agreeableness personality correlates with FoMO. Agreeableness personality is an individual with openness, like to share with others, and eagerness to want to know about others. These characters might be producing FoMO’s behavior indicator.

## Conclusions

This study is conducted in 2019 and has shown that the modified 12-item Fear of Missing Out Scale is a valid and reliable instrument for Indonesian adolescents. The results yielded a three-factor solution, and the validation can serve as the instruments in determining adolescent online behavior, mainly social media use. This study also supports the Can and Satici (2019) study that adolescents with frequent social media use have high FoMO. Specifically, the present study found that Instagram use can potentially increase the FoMO. Besides, the role of ethnicities in predicting FoMO needs further exploration. In future research, wider age groups, occupation, and comparison of social and cross-cultural context would be necessary to widen the discussion of results. The prospective study also needs to uncover the link between FoMO, internet addiction, and other psychological symptoms. Nevertheless, the increase of internet and social media use among adolescents necessitates further research to understand better the mechanism lying on internet behavior and its consequences on adolescents’ mental health. Education about healthy online behavior, interacting with other people without feeling anxious about missing out on something involving parents, and educational institution are vital to adolescents.

## Data Availability

The datasets generated during and/or analyzed during the current study are available from the corresponding author on reasonable request.

## Abbreviations

FoMO: Fear of missing out
DASS-21: Depression and Anxiety Stress Scale-21
GHQ-12: 12-item General Health Questionnaire
CFA: Confirmatory factor analysis
CFI: Comparative fit index
EFA: Exploratory factor analysis
GFI: Goodness-of-fit index
RMSEA: The root mean square error of approximation
NFI: Normed fit index
PNFI: Parsimony normed fit index
TLI: Tucker–Lewis index
